# Canine Leishmaniasis: Serological Results in Private and Kennel Dogs Tested over a Six-Year Period (2009–2014) in Abruzzo and Molise Regions, Italy

**DOI:** 10.3390/microorganisms8121915

**Published:** 2020-12-01

**Authors:** Fabrizio De Massis, Carla Ippoliti, Simona Iannetti, Manuela Tittarelli, Sandro Pelini, Daniele Giansante, Aurora Ciarrocchi

**Affiliations:** Istituto Zooprofilattico Sperimentale dell’Abruzzo e del Molise “G. Caporale” Campo Boario, 64100 Teramo, Italy; f.demassis@izs.it (F.D.M.); c.ippoliti@izs.it (C.I.); m.tittarelli@izs.it (M.T.); s.pelini@izs.it (S.P.); d.giansante@izs.it (D.G.); a.ciarrocchi@izs.it (A.C.)

**Keywords:** leishmaniasis, dogs, IFAT, survey, epidemiology

## Abstract

This paper reports the results of serological tests for the detection of antibodies against *Leishmania* spp. in Abruzzo and Molise regions from 2009 to 2014, with the aim of evaluating the presence and distribution of canine leishmaniasis. Data were extracted from the Laboratory Information Management System (LIMS) of the Istituto Zooprofilattico Sperimentale of Abruzzo and Molise, and then the dog identification numbers were matched with those stored in the Canine Registries of the two regions to get information about the age of dogs at time of testing. Dogs were considered positive when having an IFAT (Indirect Fluorescent Antibody Test) titer ≥1:80. In total, 41,631 dogs were tested, 85.3% from Abruzzo and 14.7% from Molise. At the provincial level, the percentage of positive dogs ranged from 5.2% (L’Aquila, Abruzzo region) to 21.8% (Campobasso, Molise region). Findings are consistent with the hypothesis that in the coastal areas, the relationships between the host, the vector, and the agent are more favorable for the spreading of CanL, and it seems that densely populated urban internal areas have less favorable conditions. Being a dog hosted in a kennel seems not to be a factor increasing the probability that dogs show positivity, even in long-term sheltering conditions.

## 1. Introduction

Leishmaniasis is a zoonotic disease caused by protozoan parasites of the genus *Leishmania* (Kinetoplastida, Trypanosomatidae) transmitted by the bite of phlebotomine sand flies of the genera *Phlebotomus*. About 70 species of mammals, including humans, are considered vertebrate hosts of different species of *Leishmania* spp. around the world, and some of them are reservoirs of the parasite in nature [1]. The protozoan can cause a wide variety of clinical forms ranging in severity from self-healing cutaneous leishmaniasis to fatal disseminated visceral leishmaniasis [2]. The disease is produced by the invasion of *Leishmania* spp. into the mononuclear phagocyte system of mammalian hosts and includes a group of neglected diseases that are prevalent in at least 98 countries and three territories on five continents, of which the majority are in developing countries [3,4]. In humans, leishmaniasis is second to malaria in terms of numbers of people affected worldwide [1]. Recent estimates about human leishmaniasis incidence include 12 million of people, with a ratio of 0.2–0.4 million and 0.7–1.2 million in visceral (VL) and cutaneous (CL) cases respectively, in 101 endemic countries [5,6,7].

*Leishmania infantum* is almost exclusively the main causal agent of leishmaniasis in Europe [8,9,10] and plays a critical role in both canine and human leishmaniasis, causing the VL and cutaneous CL leishmaniasis in humans, and the Canine leishmaniasis (CanL) in dogs, which is a chronic visceral–cutaneous syndrome. Infected dogs represent the main domestic reservoir of the parasite and may play a key role in the transmission to humans [11,12]. The infection is acquired when sand flies transmit the flagellated parasites into the skin of a host through the bite [2].

The existence of CanL is driven by environmental and epidemiological conditions favoring the contact between the infected sand flies and the vertebrate hosts, allowing the completion of the biological cycle of *Leishmania* spp. [4,13,14]. In Europe, both CanL and human VL are endemic in Mediterranean areas characterized by a dry, hot summer and mild winter temperatures. In particular, the disease in dogs is endemic in Portugal, Spain, France, Italy, Balkan countries, Greece, Bulgaria, and Turkey [6,15,16]. In Italy, human leishmaniasis epidemics were sporadically reported until the 1980s, and they became more frequent in the last decade with an expansion of the vectors toward northern latitudes [12,17,18]. CanL incidence has been increased in dogs since the 1990s, and classical endemic zones are the Tyrrhenian littoral, the southern peninsular regions, and the islands. Until the 2000s, stable endemic foci of both human VL and CanL were reported only in southern, central, and insular regions [19]. However, new foci of CanL and the presence of competent sand flies vectors were reported also in northern regions of the country [14]. The main vector species responsible for the disease occurrence in Italy are *Phlebotomus perniciosus* and *Phlebotomus neglectus*; other involved species are *Phlebotomus ariasi* and *Phlebotomus perfiliewi*, [15,20,21]. By comparing recent entomological surveys with the historical data available, research papers highlighted an increase in terms of population density and geographical range of *Phlebotomus perniciosus* and *Phlebotomus neglectus* from southern and central to northern Italian areas [17,22,23]. In Abruzzo and Molise, two regions of south-eastern Italy, *Phlebotomus perfiliewi* and *Phlebotomus perniciosus* have been reported respectively on the Adriatic coastal area and on the internal flat area of L’Aquila province as vectors of *Leishmania infantum* [24].

The prevention of CanL is based on the treatment of infected dogs, which may reduce the number of reservoir dogs, and the direct protection of the dogs by means of external treatment (insecticide-impregnated collars or spot-on products) with repellents and/or pesticides with biocide effect on the vector. From 2011, the possibility of vaccination to protect seronegative dogs has been made available, even if the effectiveness in protecting dogs from infection is still under discussion [2].

The aim of this paper is to report on the serological results in kennel and owned dogs tested for the detection of CanL over a six-year period (2009–2014) in Abruzzo and Molise regions of Central Italy.

## 2. Materials and Methods

### 2.1. Study Area

The study area were Abruzzo and Molise Italian regions, extending from 41°21′47″ to 42°53′48″ North latitude and from 13°0′55″ to 15°9′55″ East longitude, and facing east on the Adriatic Sea.

Abruzzo region is located on the Adriatic side of central Italy. Bordered to the east by the Adriatic Sea, Abruzzo has a 170 km long coastline. The region is characterized by the prevalence of mountainous and hilly areas: 65.1% of the regional territory is occupied by mountain systems, while the remaining 34.9% is characterized by hills that slope down from the Apennines toward the Adriatic Sea. The highest Apennine peak, being around 2912 m, named Gran Sasso d’Italia, is the natural border between the provinces of L’Aquila and Teramo. The eastern part of the region is characterized by the presence of an extended strip of hills, which are interrupted by numerous valleys in the province of Teramo and between Chieti and Pescara. The only plain is in the narrow coastal strip along the Adriatic.

Molise region is occupied by mountains (55.3%) and by hills (44.7%), for 4438 km^2^. The entire mountainous part of Molise is part of the Apennines Mountains and in particular of the southern Apennines.

### 2.2. Data Origin

Data on the sampling activities carried out on dogs tested for *Leishmania* spp. within a six-year period (from 2009 to 2014) were extracted from the Laboratory Information System (LIMS) of the Istituto Zooprofilattico Sperimentale dell’Abruzzo e del Molise “G. Caporale” (IZSAM). Data were related to dogs sampled in veterinary clinics and from public and private kennels for CanL diagnosis and control. Most data coming from veterinary clinics were related mostly to symptomatic dogs, while dogs from kennels were tested normally just after being admitted in the shelter. Given that infected dogs may have been subject to clinical follow-up during therapy, dogs having multiple positive testing in the database for the same year or in different years were considered positive when having at least one positive result (i.e., titer ≥ 1:80) in the period. The first time in life they had shown a positive result was considered as the year with positivity.

The Canine Registries of Abruzzo and Molise regions were consulted for obtaining data on the distribution of the dog population and additional information such as the date of birth. Data on human populations were obtained from the last available National Italian Census (15th Census of Population and Housing, National Institute for Statistics, ISTAT, 2011).

### 2.3. Diagnostic Methods

CanL is frequently diagnosed through the detection of specific antibodies against *Leishmania* spp., preferably using quantitative serological techniques like Immunofluorescence Antibody Test (IFAT) and Enzyme-Linked ImmunoSorbent Assay (ELISA) [2]. Sera samples submitted to the IZSAM were processed and analyzed by IFAT with antibody titers measurement. The test is considered by the International Animal Health Organization (OIE) as the reference serological method for the confirmation of clinical cases of CanL [25]. According to the laboratory Standard Operating Procedure (SOP) of IZSAM, the dogs were considered positive when the IFAT titer was ≥1:80.

### 2.4. Statistical Analyses and Software

Data from LIMS were imported in MS Access^®^ (Microsoft Access 2013, Redmond, Washington, DC, USA), which was used for cleaning and normalizing the dataset: the dog identification number (microchip or tattoo) was harmonized among its multiple occurrences (in case of multiple sampling on the same dog). Then, for the same identification number, all the negative serological results and the first positivity were kept. After the first positivity, results of further samplings were not considered.

Each identification number (i.e., each dog) was then assigned to two classes “Private” and “Kennel”, taking into account the different type of owners registered in the LIMS. In case the dogs belonged to a private owner, they were assigned to the “Private” class. In case the dogs belonged to doghouses, non-profit organization, private associations, or when the dog was registered as having a municipality as owner, the dogs were assigned to the “Kennel” class, given that these conditions would have mean that they were hosted in public or private kennels.

**Figure 1:**
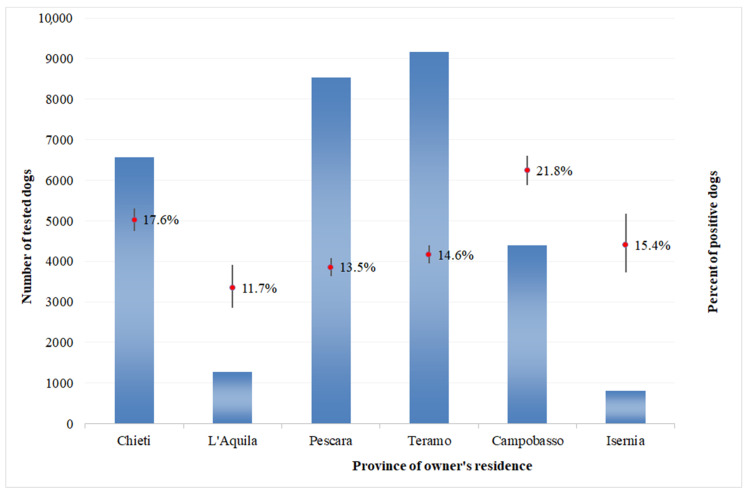
Data curation and preparation flow.

All these steps were necessary to get a dataset as accurate and consistent as possible, which is called the “cleaned database” in Figure 1. Any spurious data concerning owners from foreign regions, improbable animal species, ages, and multiple municipalities were removed from the final dataset. Concerning kennels, only the ones having collected at least 15 samples in the whole period were retained in the analysis. This has been done in order to exclude very small kennels, which could have introduced a bias in case of seropositivity recording.

Then, this dataset was matched with the Canine Registries of Abruzzo and Molise regions (data extracted on 31 December 2014) by means of the dog identification number. Only matching records were retained for further analysis. The date of birth of each animal was retrieved from the registries to calculate the age of the dog at sampling time (Figure 1).

To minimize geographical biases due to potential dog movements, the municipality of the dog was derived from the LIMS, considering that the municipality reported in the registry represented the residence at the time of data extraction from Canine Registries (31 December 2014), while the LIMS recorded data about the living place of the dog at the time of sampling.

ESRI^®^ ArcMap 10.6 (Redlands, CA, USA) software was used for geographical data management and map production. MS Excel^®^ (Microsoft Excel 2013, Redmond, Washington, DC, USA) was used for the descriptive analysis of the data.

To take into account the uncertainty of the proportion of positive laboratory results over the total tests performed, a beta distribution was used to define the 95% confidence interval of the proportion accuracy. The uncertainty interval was defined as the difference between upper and lower 95% confidence limits. The 95% lower and upper credibility levels (LCI and UCL, respectively, composing the Credibility Interval, CI) of the distribution frequency of positive results were calculated using a Bayesian approach [26] with a beta distribution β(n + 1; n − s + 1), where n is the total number of tested samples and s are the tested positive samples.

## 3. Results

In total, from 2009 to 2014, 41,631 dogs were tested, 85.3% belonging to the Abruzzo region and 14.7% belonging to the Molise region. Of the private dogs tested, 83.0% belonged to owners living in Abruzzo and 17.0% belonged to owners living in Molise. For kennel dogs, 91.6% of dogs tested were hosted in Abruzzo, while 8.4% were hosted in Molise. Overall, 73.9% of the dogs tested belonged to a private owner, and the number of private dogs tested was greater than the number of kennel dogs tested in each province, with the exception of the L’Aquila province.

At the regional level, the percentage of positive private dogs was significantly lower in Abruzzo (14.8%, CI 14.4–15.3%) than in Molise (20.8%; CI 19.7–21.9%). A similar pattern is recorded for kennel dogs, having 6.5% of positive results in Abruzzo (CI 6.0–7.0%) versus 12.8% in Molise (CI 10.8–15.1%).

The proportion of positive kennel and private dogs for each province of both regions is also detailed in Table 1.

**Figure 2:**
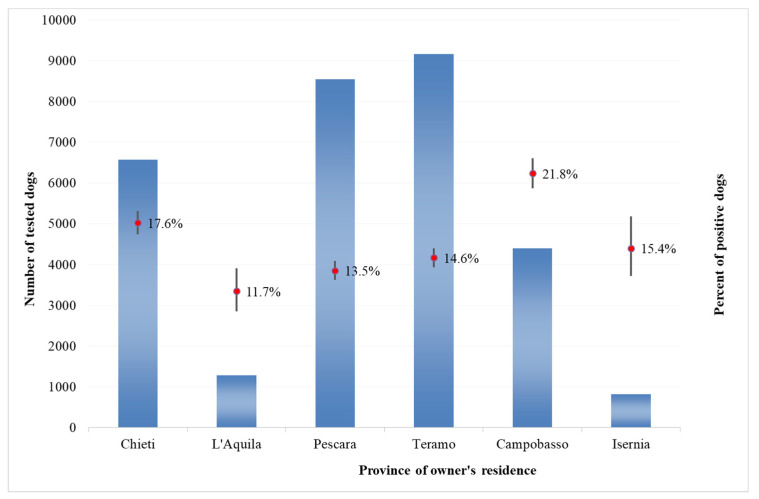
Number of private dogs tested and percentage of positive results at the provincial level in the Abruzzo and Molise regions, 2009–2014.

**Figure 3:**
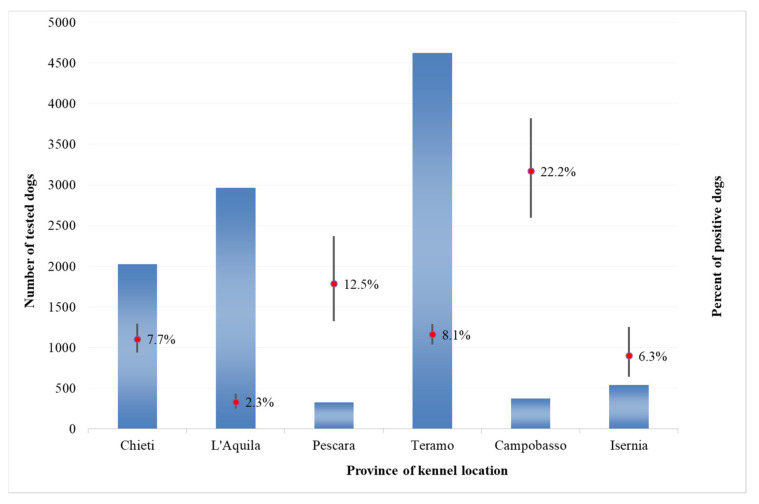
Number of kennel dogs and percentage of positive results at the provincial level in the Abruzzo and Molise regions, 2009–2014.

As regards positive results in private dogs, the percentages of dogs that tested positive were significantly higher than average in Campobasso province, Molise region (21.8%, CI 20.6–23.1%) and in Chieti Province, Abruzzo region (17.6%, CI 16.7–18.5%). This percentage was significantly lower from average for the other provinces of the Abruzzo region (Figure 2). For kennel dogs, a similar pattern has been recorded; however, percentages of positive kennel dogs are higher than average in Pescara province, Abruzzo region (12.5%, CI 9.4–16.5%) (Figure 3). In general, the number of positive dogs for CanL was higher in the group of dogs belonging to private owners, with the exception of the province of Campobasso, Molise region, where the number of positive dogs was higher in kennel dogs.

**Figure 4:**
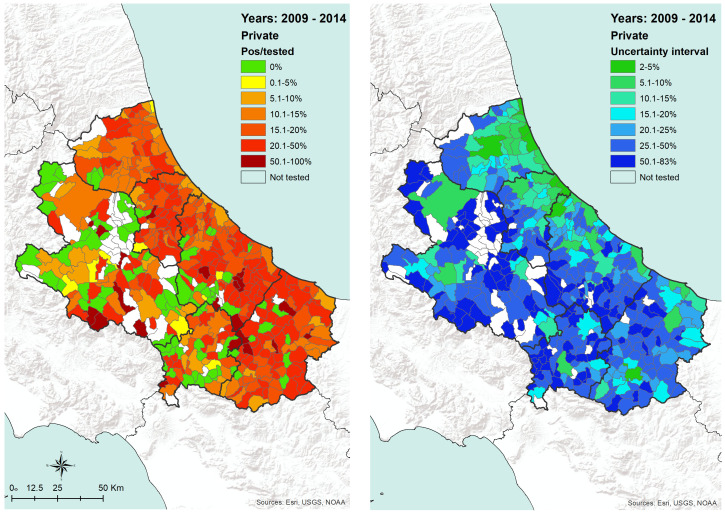
Private dogs: proportion of positive dogs tested from 2009 to 2014 and related uncertainty interval per municipality of residence at the time of sampling.

At the municipality level, the proportion of positive dogs belonging to private owners shows a geographical distribution having a higher prevalence in the coastal areas and in some municipalities in the internal territory (Figure 4, left side, which reports the proportion of dogs tested in the period considered). The uncertainty about the level of prevalence recorded in each municipality (Figure 4, right side) is lower in the most densely populated areas (green-colored in the map), where the majority of samples have been collected.

**Figure 5:**
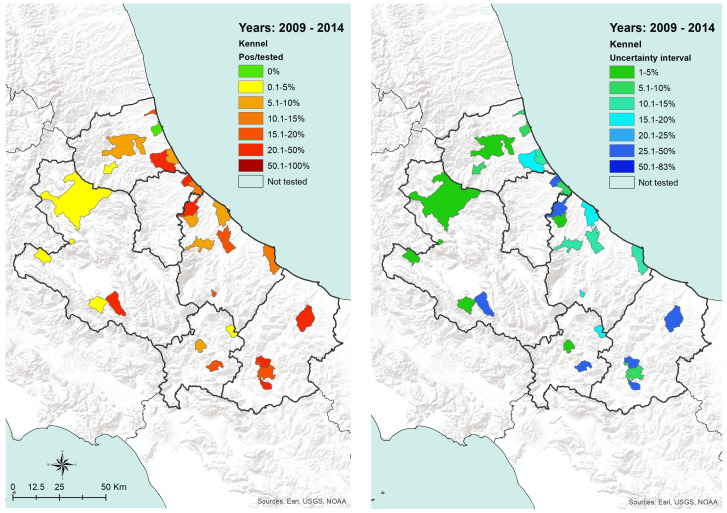
Kennel dogs: proportion of positive dogs tested from 2009 to 2014 and related uncertainty interval per municipality of kennel location.

Kennels are located only in few municipalities across the two regions. The proportion of positive dogs is scattered (Figure 5, left side), as well as the level of uncertainty about the results (Figure 5, right side).

**Figure 6:**
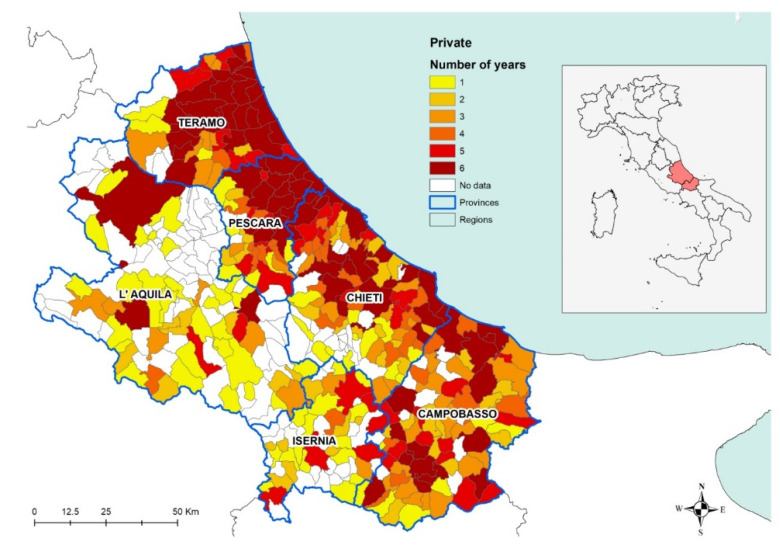
Private dogs: number of years with private dogs positive between 2009 and 2014, per municipality of residence at the time of sampling.

When analyzing the number of years from 2009 to 2014 during which positive results were recorded in private dogs, the results show how the disease is constantly present over the years in the coastal area and in the municipalities having a higher canine and human density (Figure 6). The darkest areas in Figure 6 indicate the persistence of the disease, i.e., for how many years at least one positive dog was found in each municipality in the period considered. In other words, that would be indicating the areas in which the disease has been endemic for the period considered.

**Figure 7:**
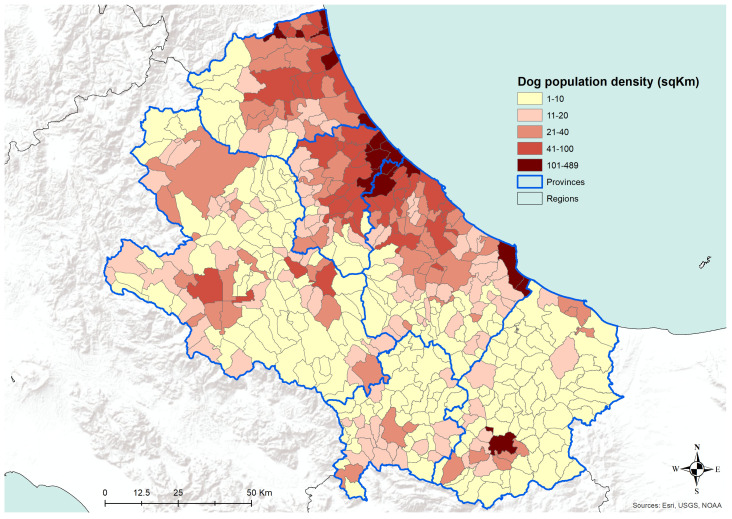
Dog population density (dog/sqKm) at the municipality level in the Abruzzo and Molise regions, 31 December 2014. Data from the Canine Registries of the Abruzzo and Molise regions.

**Figure 8:**
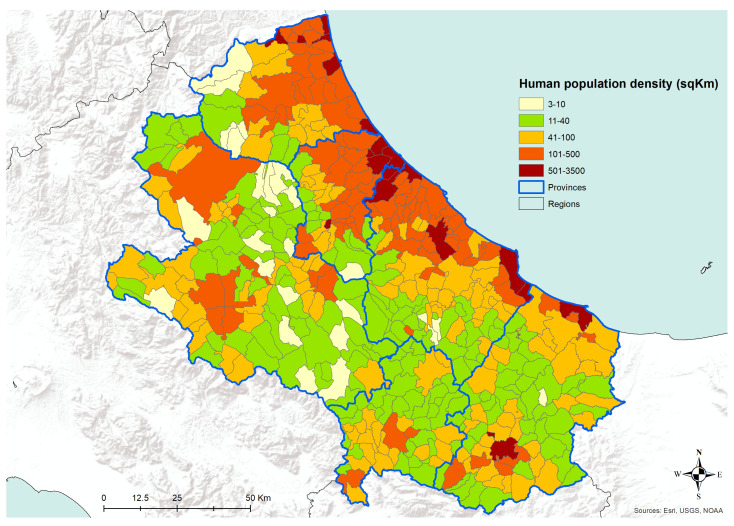
Human population density (man/sqKm) at the municipality level in the Abruzzo and Molise regions, year 2011. Data from the Italian National Institute for Statistics, ISTAT.

The analysis of the number of dogs per square kilometer, as recorded in the Canine Registries of the Abruzzo and Molise regions, has shown a pattern of higher density across the most urbanized areas (Figure 7). The density of registered dogs is higher in the municipalities in the coastal areas and in some municipalities in the internal territories. A similar pattern is confirmed by the analysis of human population density data, as derived from the National Institute for Statistics, ISTAT (Figure 8). Actually, in Figure 8, the municipalities showing the higher human population density are the major cities of the two regions, i.e., cities having more than 15,000 inhabitants.

**Figure 9:**
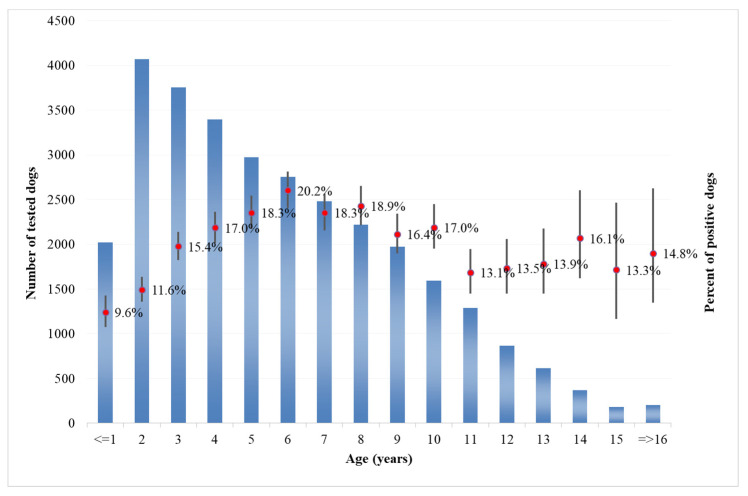
Distribution of tested (blued bars) and positive (red point and confidence bars) private dogs by age classes.

**Figure 10:**
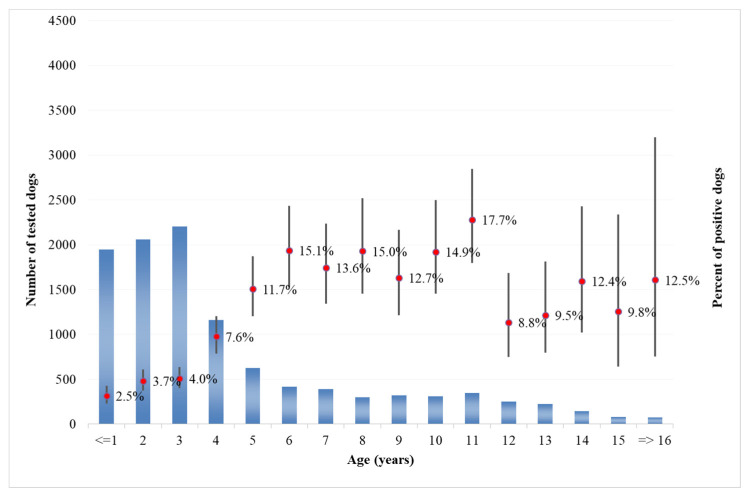
Distribution of tested (blued bars) and positive (red point and confidence bars) kennel dogs by age classes.

Analysis of the age of dogs at the time of sampling showed that in both categories, the majority of tested dogs were from 1 to 10 years old (Figure 9 for private owner, Figure 10 for kennels). The number of positive dogs is increasing with the age at sampling, as expected. Interestingly, in kennel dogs, the percentage of positive dogs is significantly lower than the percentage recorded in owned dogs for young animals.

## 4. Discussion

This study represents the first attempt aimed at discussing the results of six-year (2009–2014) monitoring activities on CanL to have insights for better understanding the distribution of the disease in dog populations over the two regions concerned. To the best of our knowledge, the data analyzed are the only study of CanL diffusion available at the municipality level in Italy, and the only study carried out on an important database, which accrued data from 30,776 samples from private dogs and 10,855 samples from dogs hosted in kennels. The large number of collected data gives solidity and consistency to the results obtained and thus the analysis made.

As far as private dogs are concerned, the results reveal that during that period, CanL has been continuously threatening private dog populations in the territory under study (Figure 6), confirming its status as endemic disease in the areas under study. In particular, the burden of the disease appears to be higher in the coastal area, recording a stable number of positive dogs all across the years considered. Conversely, seropositivity spots are recorded in the internal (mostly mountainous) areas, corresponding with the most populated municipalities, which are also the most populated dogs areas. This is confirmed by the low uncertainty of the results obtained in coastal areas in particular and in densely populated areas in general (Figure 4).

The transmission of CanL is complex due to the varying ecological relationships between human and/or animal reservoir hosts, parasites, and sand fly vectors. Moreover, vector-borne diseases such as leishmaniosis are intricately linked to environmental changes and socioeconomic risk factors, advocating the importance of the One Health approach to control these diseases [27]. Our findings are consistent with the hypothesis that in the coastal areas, the relationships between the host, the vector, and the agent are more favorable for the spreading of CanL, as well as it seems in densely populated urban areas, even in the internal mountainous and colder territories. The coastal areas are those where the density of dogs and humans are higher (Figure 7 and Figure 8), and this is probably related to an historical higher economical capacity of the inhabitants. They are more prone to hold pets, thus increasing the density of the hosts and, as a consequence, of the related disease vectors. This is confirmed by the finding that rural areas and the mountainous part of the territory are found free from the disease or seem to suffer a lower impact, except for bigger municipalities. In these latter territories, less favorable conditions for the vectors would be expected; however, they seem not to eliminate the vector capacity to infect new canine hosts, at least in densely urbanized areas. However, the low number of controls performed gives a higher uncertainty level for the results recorded in the internal areas (Figure 4 and Figure 5). Actually, in many of the municipalities of the internal territories, the sample size was low (if not zero), and this may have led in some cases to an overestimation or an underestimation of the prevalence. This is particularly evident in some municipalities located in mountainous or less urbanized areas where it is likely that the sensitivity toward prevention and screening tests is lower, and it is possible that the few samples received were based only on clinical suspicion. Another factor to consider is the orography of the province; in fact, in the mountainous areas, which represent more than half of the territory, the canine population has a very low density if we exclude the big cities. However, the uncertainty of the results obtained from the internal territories (Figure 4 and Figure 5) could be reduced only by increasing in the future the levels of testing.

Changes in the environment also have a strong impact on leishmaniosis, either due to climate change caused by global warming or man-made ecological alterations. An abrupt climate change exerts an influence on the migration of sand fly vectors and reservoir animals [27]. Numerous studies have shown that leishmaniasis disease distribution has been shifting and will shift as a response to climate change [28]. As far as the spatial and temporal dynamics of the disease in the study area are concerned, positive results are recorded in each of the sampling years of the period considered mainly in the municipalities of the coastal areas (in space and time). However, it is difficult to identify factors related to climate change or change in the urbanization pattern in the short period studied (6 years), even if other studies have already shown that a sudden climate change influences leishmaniasis disease distribution [27,29].

**Figure 11:**
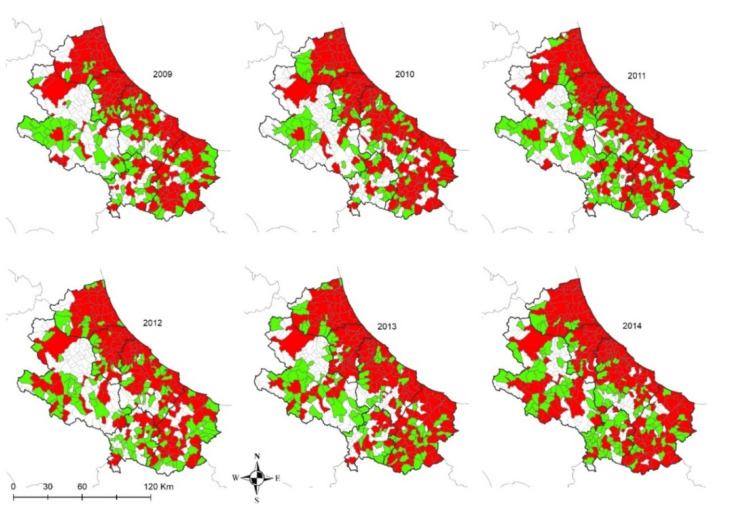
Private dogs: municipalities with *Leishmania* spp. positive (in red) and negative (in green) dogs from 2009 to 2014 in Abruzzo and Molise regions, per municipality of residence at the time of sampling.

**Figure 12:**
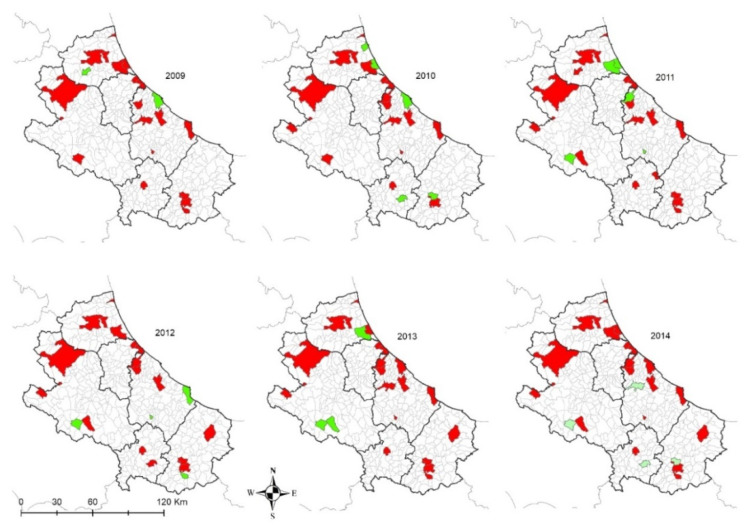
Kennel dogs: municipalities with *Leishmania* spp. positive (in red) and negative (in green) dogs from 2009 to 2014 in Abruzzo and Molise regions, per municipality of kennel location.

In the internal areas, the distribution of the disease appears to be more erratic during years, however, if we consider dogs hosted in densely populated human-dog areas (Figure 4 and Figure 5), the temporal pattern is consistent with that recorded in the coastal areas (Figure 11 and Figure 12) [30,31]. Data show a similar pattern between dogs and human population densities, and this may suggest that the two densities are running in parallel and so may occur for the risk of transmission of CanL at the dog/human interface (Figure 4 and Figure 5).

The percentages of dogs that tested positive were significantly higher than average in Campobasso province, Molise region (21.8%, CI 20.6–23.1%) and in Chieti province (17.6%, CI 16.7–18.5%). These are neighboring provinces and are the most southern of the two regions, suggesting the existence of common spreading factors and conditions more favorable to the vectors development (such as favorable climate). However, the impact of this variable needs to be further investigated.

As regards the age distribution of positive private dogs (Figure 9), the data are confirming findings reported in other papers, where age has been positively associated with seropositivity, i.e., older ages has been identified as a predisposing factor for the development of antibodies against *Leishmania* spp. [32]. As far as kennel dogs are concerned, the results reveal that in the period under study, the majority of investigated kennels show at least one positivity in dogs hosted (Figure 12). The results have a lower uncertainty level in areas where kennels are more populated and in which a higher number of samples have been collected during years (Figure 5). This can be consistent with the fact that normally kennels are hosting stray dogs in, i.e., dogs possibly already infected when entering the kennel. Giving that the leismaniasis test is compulsory in Italian kennels, these positive results would be revealed on the date of their confinement. This finding could also be discussed with the hypothesis that since kennels often host dogs for long periods (long-term sheltering), the probability that their hosts had the infection already circulating in the area may be increased by such condition. However, this study reveals a significantly lower percentage of kennel positive dogs belonging to younger age classes (Figure 9 and Figure 10) with respect to private dogs.

The results show that the percentage of positive dogs is significantly higher in private dogs rather than in kennel dogs for the ≤1–9 years age class, and that the results are not significantly different in higher age classes (Figure 9 and Figure 10). This finding may support the consideration that in the areas concerned, being a dog hosted in a kennel is not a factor increasing the probability that animal will show positivity, even if in long-term sheltering conditions.

As regards control and surveillance of CanL in Italy, there is no legal requirement for CanL, except for Article 5 of the Veterinary Book of Rules on Animal Health, which obliges the veterinary services and the health services to exchange information about the occurrence of zoonosis both in animals and men, including CanL [33]. Specific monitoring plans for the control of CanL have been put in place in some regions [34]. These plans aim at monitoring human VL and CanL through a constant and targeted verification of the presence of the disease in dogs with owners and stray dogs, and at identifying control measures to be adopted in relation to the affected dogs. For instance, in Campania, the regional monitoring plan prescribes that in endemic areas (identified as the geographical areas in which a high prevalence of CanL and recurrent cases of human VL have been recorded in the past), dog owners must submit their dogs annually to clinical examination and blood sampling for the diagnosis of CanL. Other regions that do not have specific monitoring plans in place apply the legislation prescribed in the Veterinary Book of Rules on Animal Health. This is the case of the Abruzzo and Molise regions, where the public health veterinarians are in charge of controlling CanL only on kennel and stray dogs through annual serological testing [34,35]. Private dogs are usually tested in case of clinical signs attributable to the disease (suspect sampling), with a request made to the laboratory by private practitioners. Dogs can be also tested in the context of research projects (surveys) involving both private and kennel/stray dogs. Moreover, the regional legislation of Abruzzo provides monitoring for the serological rate of the leishmaniasis, but it does not suggest what provisions must be taken in case of seropositivity.

Although human leishmaniosis represents a rare parasitic disease in man in European countries, it is endemic in dogs in many countries, and particularly in the Mediterranean basin, including Italy.

CanL is historically endemic in Italy in center–south regions and in the islands. Nowadays, the disease is endemic in all the Tyrrhenian regions, in those of the Southern Adriatic Sea, and as well in the islands. During the years, the country-wide incidence of CanL has been increasing, with new foci being detected within traditional boundaries of endemic transmission but also in northern regions previously regarded as non-endemic [17,18,36]. Seroprevalence rates reported until now by several authors are different and discordant, depending on the criteria adopted for sampling: if tests are performed on sick dogs, the percentage will clearly be higher than screening done according to a random system. As discussed previously, the differences related in seroprevalence to various altitudes, from the climate on the coast to the climate in the mountains, must be taken into consideration. This highlights the need for adequate monitoring, including the harmonization of notification at any decision level, from local villages to among countries, and a comprehensive set of strategies aimed at reducing the burden of the disease in target populations. This would benefit from studies on the ecology of the disease both in humans and in animals, strengthening the research on the determinants of the disease at the animal–human interface.

## Figures and Tables

**Table 1:** Proportion of the studied private and kennel dogs that tested positive (P%) in Abruzzo and Molise regions from 2009 to 2014.

Region	Province	Private Dogs			Kennel Dogs		
		N. Tested	N. Positive	P% (±95% CI)	N. Tested	N. Positive	P% (±95% CI)
**Abruzzo**	Chieti	6572	1155	17.6 (16.7–18.5)	2027	157	7.7 (6.7–9.0)
	L’Aquila	1280	150	11.7 (10.1–13.6)	2965	69	2.3 (1.8–2.9)
	Pescara	8542	1152	13.5 (12.8–14.2)	328	41	12.5 (9.4–16.5)
	Teramo	9164	1336	14.6 (13.9–15.3)	4622	376	8.1 (7.4–9.4)
**Molise**	Campobasso	4400	960	21.8 (20.6–23.1)	374	83	22.2 (18.3–26.7)
	Isernia	818	126	15.4 (20.6–23.1)	539	34	6.3 (4.6–8.7)
**Total**		30,776	4879	15.9 (15.4–16.3)	10,855	760	7.0 (6.5–7.5)
